# Two new records of the fern genus *Coniogramme* (Pteridaceae) from Vietnam

**DOI:** 10.3897/phytokeys.119.33126

**Published:** 2019-04-11

**Authors:** Caihong Wang, Wenli Yang, Junwen Zhao, Danke Zhang, Gangmin Zhang

**Affiliations:** 1 Laboratory of Systematic Evolution and Biogeography of Woody Plants, College of Nature Conservation, Beijing Forestry University, Beijing 100083, China Beijing Forestry University Beijing China; 2 College of Landscape Architecture and Tourism, Hebei Agricultural University, Baoding 071000, China Hebei Agricultural University Baoding China

**Keywords:** Cryptogrammoideae, taxonomy, Vietnam

## Abstract

Two new records of the fern genus *Coniogramme* Fée from Vietnam, *C.japonica* and *C.procera*, are presented. In addition, a key to recognising the species of *Coniogramme* in Vietnam is given in this paper.

## Introduction

*Coniogramme* Fée, which belongs to the subfamily Cryptogrammoideae in the family Pteridaceae (Christenhusz et al. 2011; Zhang and Ranker 2013; PPG I 2016), is mainly distributed in the tropical and subtropical regions of Asia, extending south to Africa. It is characterised by its large habit with creeping rhizomes and 1–3 pinnate fronds with exindusiate sori borne along the lateral veins.

The genus was first monographed in its modern form by Hieronymus (1916), who accepted 17 species in the world. Dixit and Das (1979) recorded 13 taxa within the genus from India. In the course of the study of pteridophytes in China, some scholars (Ching 1930, 1974, 1982; Shing 1981; Kong 1982; Ching and Liu 1984; Guo and Chen 2013) have published more than 30 new taxa of *Coniogramme*. Amongst them, Shing (1981) reported 27 new taxa and recognised 39 species and eight varieties in Flora Reipublicae Popularis Sinicae (Shing 1990). In the taxonomic keys, many species are distinguished only by the shape of pinnules, size of serrated teeth from pinnule margins and position of hydathodes at the top of the veins, but these traits are unstable and vary occasionally within normal populations and are difficult to use in practical identification. This taxonomic treatment has been described as the “inclusion of many erroneous new species entirely confusing the variation within species which is easily observable in the field” (Fraser-Jenkins et al. 2015). So far, the genus has long been one of the most problematic fern groups with respect to its specific definition. Fraser-Jenkins (2008) concluded that the taxonomy of *Coniogramme* is very complicated and has been confusing taxonomists in species circumscription.

Although many new taxa have been recorded recently, the fern diversity in Vietnam remains unclear (Lu et al. 2014). In revising the taxonomy of *Coniogramme*, the senior author was fortunate to visit Vietnam and examine the specimens deposited at HNU and HN. Two new records were discovered, i.e. *C.japonica* (Thunberg) Diels and *C.procera* Fée. This work is a contribution to the knowledge of fern flora in Vietnam.

## Results

### Coniogramme japonica

Taxon classificationPlantaePolypodialesPteridaceae

(Thunb.) Diels (1899: 262)

Figures 1 , 3

#### Type.

Japan. No exact location. *C. P. Thunberg s.n.* (UPS!).

#### Specimens examined.

**Vietnam. Cao Bang Province**: Ha Lang District, Dong Loan municipality, vicinities of Ban Lung and Lung Phuc, 22°46'N, 106°44'E, 500–600 m elev., 25 Nov 1998, *L. Averyanov et al. CBL 656* (HN). **Bac Kan Province**: Cho Don District, Ban Thi municipality, Phia Khao village, 22°17'03"N, 105°30'34"E, ca. 800 m elev., 5 Mar 2011, *N.Q. Hieu, N.T. Hiep, P.K. Loc, P.V. The, & N.T. Vinh CPC 1240* (HNU 017603, HNU 017604).

**Figure 1.**
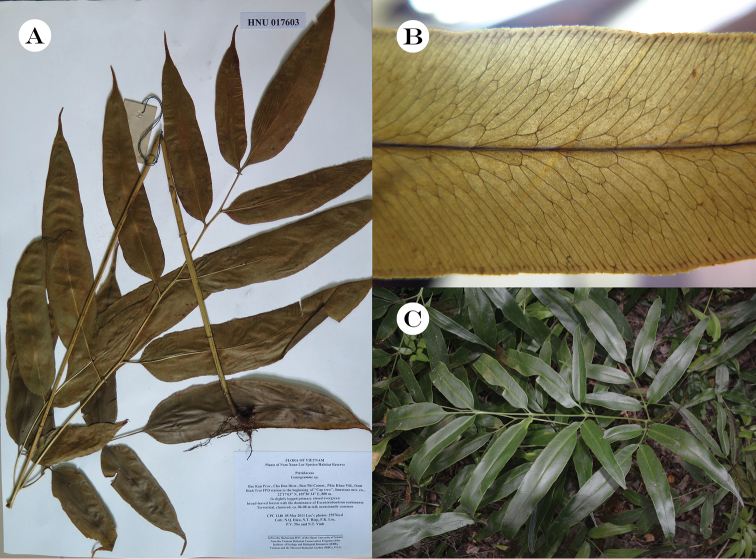
*Coniogrammejaponica* (Thunberg) Diels **A** one of the voucher specimens **B** portion of a pinnule, showing anastomosing veins **C** morphology and habitat.

#### Taxonomic notes.

This species is very unique in morphology, differing from other species in its anastomosing veins, which form 1–3 rows of areoles along each side of midrib and hydathodes not extending the base of short serrated teeth. Shing (1981, 1990) considered that pinnules of *Coniogrammejaponica* are narrowly lanceolate with a cuneate or rounded-cuneate base and published a similar new species, *C.centrochinensis* Ching, whose pinnules were widely lanceolate with a rounded base. After specimen examination, combined with fieldwork, we found that the morphology of the pinnules was not stable and varied occasionally within normal populations. More research work should be undertaken to elucidate their phylogenetic relationship.

#### Distribution and habitat.

*Coniogrammejaponica* is distributed in China, Japan (including Ryukyu Islands), Korea and Vietnam (new record). The species usually grows in shady wet places at an elevation of about 100 to 2000 m.

### Coniogramme procera

Taxon classificationPlantaePolypodialesPteridaceae

Fée (1865: 22)

Figures 2 , 3

#### Type.

Nepal. April 1821. *Wallich no 3* (K!).

#### Specimens examined.

**Vietnam. Kon Tum Province**: NW slopes of Ngoc Link mountain, 2380 m elev., 06 Mar 1995, *L. Averyanov et al. VH 519* (HN); W slope of Ngoc Link mountain, 1950 m elev., 10 May 1995, *L. Averyanov et al. VH 1290* (HN).

#### Taxonomic notes.

This species is large and up to 1.8 m tall, differing from other species in its far more dissect laminae, basal pinnae having more than 10 pairs of pinnules, pinnules with rounded-truncate or truncate (sometimes slightly cordate) base and coarsely serrated margin and sori extending only to 1/2–2/3 of veins. Fraser-Jenkins (2008) reported that the species has a characteristically strong odour when the leaves were crushed or broken, similar to that of *Coniogrammefraxinea* (D.Don) Diels.

**Figure 2.**
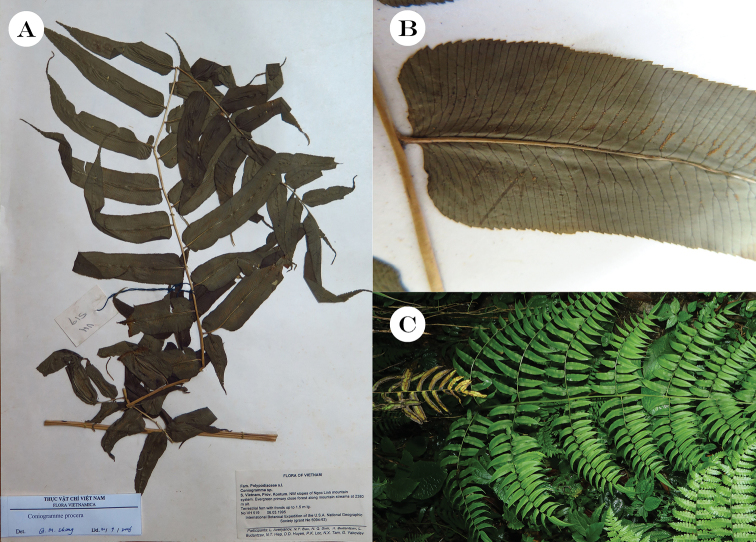
*Coniogrammeprocera* Fée. **A** One of the voucher specimens **B** Portion of a pinnule, showing a truncate base **C** Morphology and habitat.

**Figure 3.**
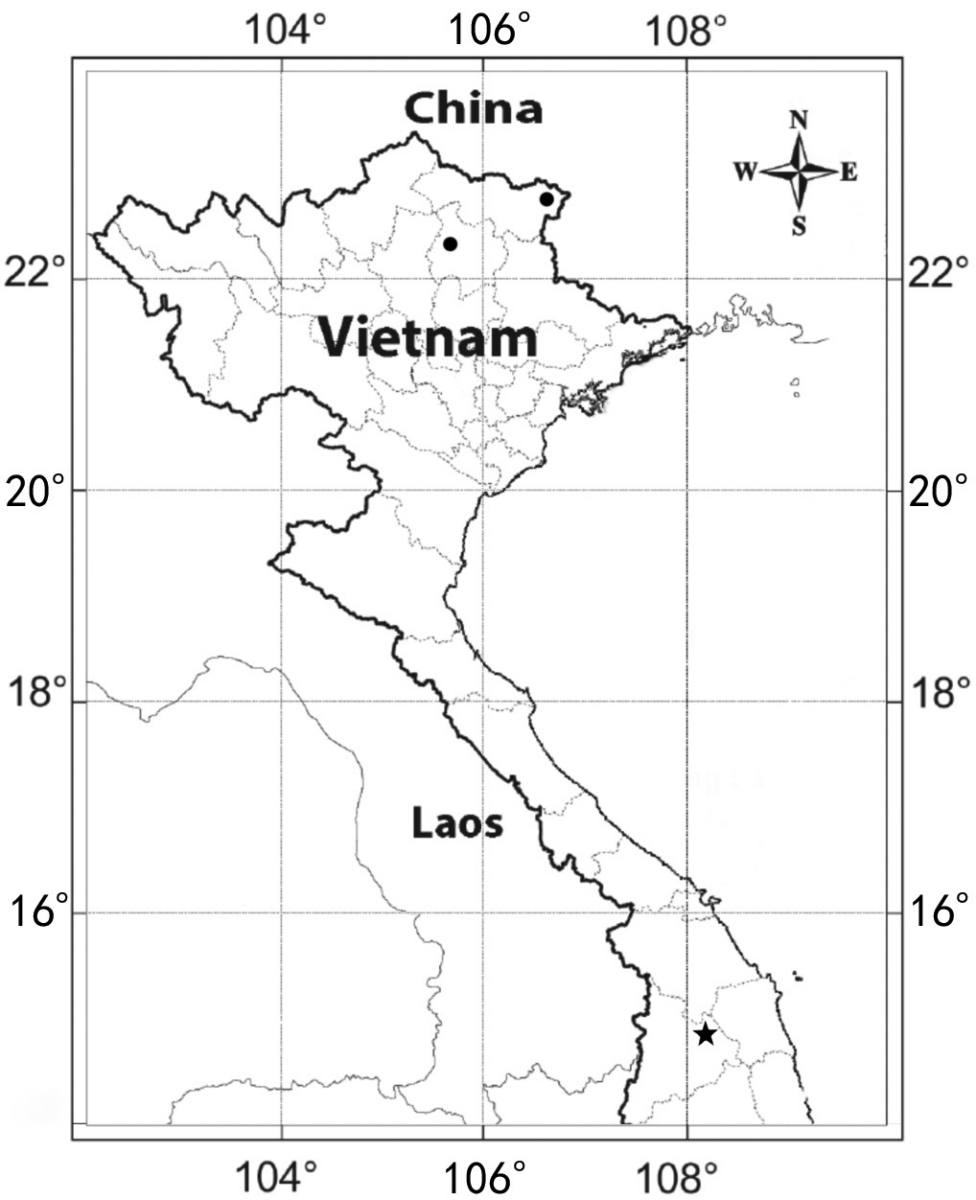
Distribution of *C.japonica* (dots) and *C.procera* (star) in Vietnam.

#### Distribution and habitat.

*Coniogrammeprocera* was once recorded being distributed in Vietnam in Flora Reipublicae Popularis Sinicae (Shing 1990) and this was followed by Flora of China (Zhang and Ranker 2013). After specimen examination, we found that there were no accounts of *C.procera* in K, BM, P, PE and other major herbaria and we wondered about the basis of this recognition. In addition, the species has never been recorded in the literature on flora of Vietnam (Tardieu-Blot and Christensen 1941; Pham 1991; Phan 2010), including the recently updated checklist (Phan 2010). *C.procera* is therefore confirmed to be distributed in central Vietnam for the first time. It is also distributed in Bhutan, China, India, Myanmar, Nepal, Philippines and Thailand. The species usually grows by streams in woodlands at a high elevation, about 1400 to 3600 m.

Based on previous literature (Tardieu-Blot and Christensen 1941; Pham 1991; Phan 2010), along with our specimen identification work at HNU, HN and K, six taxa of *Coniogramme* were recognised in Vietnam, namely *C.fraxinea* (D.Don) Diels, *C.intermedia* Hieron., *C.macrophylla* (Blume) Hieron., *C.petelotii* Tardieu, *C.japonica* (Thunberg) Diels and *C.procera* Fée. Their main differences in character were illustrated in the following key:

### Key to the species of *Coniogramme* in Vietnam

**Table:** 

1	Veins anastomosing to form 1 or 2 continuous rows of areoles on each side of midrib	*** C. japonica ***
–	Veins all free	**2**
2	Pinnule margins entire	**3**
–	Pinnule margins serrate	**5**
3	Hydathodes extending to cartilaginous lamina margin	*** C. macrophylla ***
–	Hydathodes spindle-shaped, not extending to lamina margin	**4**
4	Base of pinnules rounded or slightly cordate	*** C. petelotii ***
–	Base of pinnules cuneate or rounded-cuneate	*** C. fraxinea ***
5	Basal pinnae having more than 10 pairs of pinnules; pinnules broadly lanceolate, base rounded-truncate or truncate (sometimes slightly cordate)	*** C. procera ***
–	Basal pinnae having 2–3 pairs of pinnules; pinnules lanceolate, base rounded to rounded-cuneate	*** C. intermedia ***

## Supplementary Material

XML Treatment for Coniogramme japonica

XML Treatment for Coniogramme procera
